# Alcohol Consumption of Male Tuberculosis Index Cases and Tuberculosis Transmission Among Social Contacts in Puducherry, India: A Cross-Sectional Analytical Study

**DOI:** 10.3390/tropicalmed10090248

**Published:** 2025-08-30

**Authors:** Charutha Retnakumar, Palanivel Chinnakali, Balaji Bharadwaj, Karikalan Nagarajan, Sonali Sarkar

**Affiliations:** 1 Department of Preventive and Social Medicine, Jawaharlal Institute of Postgraduate Medical Education and Research, Puducherry 605006, India; dr.charutha.r@gmail.com (C.R.); palaniccm@gmail.com (P.C.); 2 Department of Psychiatry, Jawaharlal Institute of Postgraduate Medical Education and Research, Puducherry 605006, India; drbalaji.jipmer@gmail.com; 3 Department of Behavioural Research, Indian Council of Medical Research, National Institute for Research in Tuberculosis, Chennai 600031, India

**Keywords:** latent tuberculosis infection, alcohol drinking, social network, contact tracing

## Abstract

We aimed to compare the proportion of tuberculosis infection among social contacts of male tuberculosis Index case with and without alcohol use in the Puducherry district. A cross-sectional study using ego-centric approach was conducted between November 2023 and May 2024. A total of 713 social contacts of 106 male pulmonary tuberculosis index cases were enrolled, stratified by alcohol-use (AUDIT ≥ 8): 358 contacts from 45 alcohol-using cases and 355 from 61 non-alcohol-use cases. Social contacts were defined based on the frequency and duration of shared indoor exposure with index cases within the past three months. Tuberculosis infection was screened with Cy-Tb skin test (≥5 mm induration) at the third month of index case treatment. Univariate and multivariable analysis were conducted to identify factors associated with tuberculosis transmission. Among the 358 social contacts of alcohol-use index cases, 33.8% (*n* = 121; 95% CI, 29.1–38.8%) tested positive for tuberculosis infection, significantly higher than 21.7% (n = 77; 95% CI, 17.7–26.3%) among 355 contacts of non-alcohol-use cases. Regression analysis revealed that contacts of alcohol-using index cases (aOR = 1.6, *p* < 0.05), were significantly associated with tuberculosis infection. Alcohol-use among tuberculosis patients significantly increases the risk of tuberculosis infection in their social networks.

## 1. Introduction

The National TB Elimination Program of India, aims for tuberculosis (TB) elimination by 2025 which is unmet and remains a substantial challenge [1]. While there is substantial progress in terms of reducing TB incidence by 17.7% since 2015, still the national target of reducing TB incidence by 80% remains elusive [2]. This challenge is underscored by the fact that 5–10% of those with tuberculosis infection (TBI) will develop active TB during their lifetime [3]. To halt the progress of TBI to active disease, it is crucial to screen and treat TB infection, especially among contacts of active TB cases [4]. To lower TB incidence, it is essential to break the chain of transmission [5].

Alcohol plays an important role in TB transmission. However, the mechanisms remain poorly understood. It is a known risk factor for TB, not only in terms of susceptibility but also in facilitating transmission [6]. It weakens immunity, increases infection risk, linked to poor treatment adherence and increased social interaction in crowded, poorly ventilated-spaces like drinking venues [7]. Alcohol use disorders (AUD) are highly prevalent among persons with TB (PTB) in India [8]. They often spend more time in social settings, thus increasing the risks of transmission to their social contacts (SC) [9]. Non-household contacts, often neglected, may significantly contribute to transmission, particularly in socially active settings like alcohol consumption. Though household transmission matters, studies suggest that a large proportion of transmission occurs in community settings [10,11]. Social interactions, including close contact in neighborhoods, workplaces, and closed settings like alcohol-serving venues, drive TB transmission [12,13,14,15].

Contacts of PTBs are at higher risk of infection than the general population. The risk of TBI depends on the individual’s immunity, the patient’s infectiousness (e.g., sputum smear positivity), proximity, and duration of exposure [16,17]. Systematic screening of high-risk groups and close contacts of patients with TB disease, is one of the cornerstones of the End TB approach [18]. The key to the prevention of TB is tracing and investigating contacts of PTBs. Until recently, India’s TB programme focused mainly on household contacts. On the 7 December 2024, India launched the 100-day TB campaign aiming to provide TB preventive treatment to vulnerable populations, such as smokers, alcohol users, the elderly, those with past TB, the malnourished, and individuals living with HIV in 347 high-burden districts [19]. However, the alcohol users and their contacts remain hard to reach [20]. Social contacts of index cases were usually overlooked in routine TB contact tracing, mainly because of the stigma related to both TB and alcohol use. Healthcare workers often find it repugnant to conduct screening in settings such as arrack shops. Moreover, index cases are often reluctant to share details of their social networks, making these contacts difficult to identify and test [21]. Social contacts commonly belong to marginalized groups, such as daily wage laborers with alcohol use habits, who have limited access to health services and are frequently on the move for work, complicating tracing efforts [22]. As a result, national tuberculosis programs often focus on household contact investigations and do not routinely offer screening in such difficult to reach social networks. Understanding TB infection among these social contacts is crucial, as they represent an important but unrecognized route of transmission. This study addresses this gap by comparing the burden of TBI among social contacts of pulmonary TB patients with and without AU in the Indian setting.

Puducherry, a Union Territory in southern India, has a high TB burden and a higher prevalence of alcohol-use among PTBs than other high TB burden areas within India. Previous study shows that in Puducherry, 59% of PTBs consumed alcohol, and 54% of them had AUD based on the Alcohol Use Disorders Identification Test (AUDIT) [23]. AU in this setting is predominantly found in males. In this study, we aim to compare the proportion of TBI among social contacts of male PTBs with and without AU in the Puducherry district.

## 2. Materials and Methods

### 2.1. Study Design, Population and Setting

A community-based, cross-sectional analytical study was undertaken in the Puducherry district between November 2023 and May 2024 to identify TBI among social contacts of male pulmonary PTBs, stratified by alcohol consumption. The study population consisted of social contacts of men with microbiologically confirmed pulmonary tuberculosis receiving treatment in the district. Women were excluded to ensure homogeneity, as AU is less common among them.

The Government Chest Clinic (GCC) in Puducherry which is the district’s central TB registry, provided the list of newly reported male pulmonary TB cases. Patients were contacted through their designated Primary Health Centers (PHCs) and enrolled at a convenient time and place, either at the PHC or their residence, based on their preference.

### 2.2. Data Collection Tools

Following written informed consent, each index case (IC) was interviewed using a semi-structured questionnaire adapted from a similar study conducted in Chennai [24]. The questionnaire had undergone internal validation and was piloted prior to data collection to ensure contextual appropriateness in Puducherry. No modifications to the questionnaire were necessary, as the study population shared cultural and geographic similarities with that of a previous study conducted in Chennai, Tamil Nadu, which is approximately 100 miles from Puducherry. Alcohol use was assessed using the AUDIT tool [25], with a score ≥ 8 used to classify participants into alcohol-use and non-alcohol-use groups.

The study used an egocentric approach, relying on each index case to nominate their social contacts. Index cases reported individuals they had shared enclosed spaces with, such as in neighborhoods, workplaces, public venues, or drinking settings, during the three months before diagnosis. Pregnant women, children under one, and anyone who had recently received the BCG vaccine were excluded.

Information on socio-demographic characteristics, medical history, behavioural risk factors such as alcohol use, tobacco use, TB-related parameters, and anthropometric measurements were collected via face-to-face interviews. Contact and venue-level exposures were captured.

To assure data quality, each participant was allocated a unique ID to maintain confidentiality and facilitate the linkage of index and contact data. To mitigate recall bias, memory cues (including festivals, travel, hospitalisation, social gatherings, and key events such as marriage, funeral etc.) were employed to aid participants in recollecting timelines and contacts. Key questions were repeated in several to validate responses.

### 2.3. Screening for TBI

**Figure 1:**
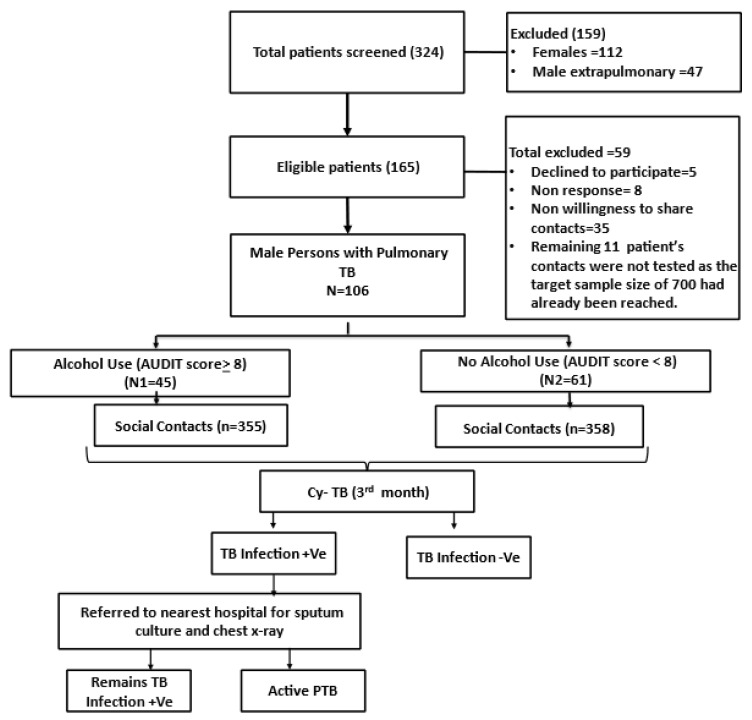
TB screening and follow-up pathway among social contacts of TB index cases with and without alcohol use.

Cy-Tb skin test was administered intradermally to the contacts at the 3rd month of index cases treatment. An induration of ≥5 mm was considered TBI positive. After evaluation for TBI, the contacts were referred to the nearest hospital or government chest clinic for chest x-ray and sputum testing to rule out active TB (Figure 1).

### 2.4. Operational Definition

- Persons with pulmonary tuberculosis (PTB)/Index case (IC): Persons with confirmed tuberculosis by sputum smear microscopy/CBNAAT/Gene-Xpert.
- Alcohol use: Defined as study participants who scored ≥8 when screened using the Alcohol Use Disorder Identification Test (AUDIT).
- Social contact (SC): Non-household contact who shared an enclosed space (e.g., at social gatherings, workplaces, or other facilities) with the index case for at least three days per week, for two to four hours per day, in the three months preceding the index case’s current treatment episode.
- Casual and close contact: Based on the duration of time spent with the index case, social contacts were categorized as casual or close using a weighted score from three factors:
  - Time spent with the index case (<4 weeks = 1, 4–8 weeks = 2, >8 weeks = 3);
  - Frequency per week (3 or more times/week = 1, daily = 2);
  - Hours per week (2–4 h = 1, 4+ h = 2, all day = 3).
    ○ Those with a total score ≥ 6 was classified as close contacts, and those with a score <6 as casual contacts. A data-driven methodology for classification was used, were a score of 6 was determined based on the mean score obtained from the study population.
- ***Tuberculosis infection (TBI):*** A person who undergoes Cy-TB testing and develops an induration of 5 mm or more is considered to have TB infection.

### 2.5. Ethics

Ethical approval was obtained from the Institutional Ethics Committee (IEC) of JIPMER, and administrative clearance from the State TB Control Officer, National Tuberculosis Elimination Programme (NTEP), Puducherry.

### 2.6. Sample Size

A sample size of 314 per group was calculated for this study using a 10% difference in the prevalence of TBI, based on an assumed prevalence of 31% among contacts of PTBs with AU and 21% in the other group as per the findings of the National TB Prevalence Survey India 2019–2021 [26], with a 95% significance level and 80% power. Accounting for a potential 10% non-response among contacts, the target sample size was 700 (350/group).

### 2.7. Study Participants

Of 324 pulmonary TB patients screened, 159 were excluded (112 females and 47 with extrapulmonary TB). Among the remaining 165 patients, 48 declined participations, did not respond, or were unwilling to share contacts and 11 additional patients were not enrolled because the sample size of 700 contacts had already been achieved by enrolling 106 patients. A total of 106 index cases were enrolled: 45 with alcohol use (AU) and 61 with no alcohol use (NAU). These index cases reported 994 social contacts (476 from AU and 518 from NAU), of which 878 were identified and 713 were tested (358 in AU and 355 in NAU). Exclusions among contacts included 7 pregnant contacts and 79 who refused testing (31 in AU and 48 in NAU). We did not halt testing midway upon reaching 700, as it was important to test all contacts of the enrolled patients for completeness. All contacts listed by the included patients were tested, resulting in a total of 713 contacts.

### 2.8. Statistical Analysis

Descriptive statistics summarized the baseline characteristics of the participants, including proportions for categorical variables and means with 95% confidence intervals (CIs) for continuous variables. Chi-square tests were used to compare the proportion of TBI between groups. Univariate analysis was performed to assess the association between each explanatory variable and the TBI in social contacts. Explanatory variables with a *p*-value ≤ 0.2 In the univariate analysis were included in a multivariable logistic regression model to identify the association of AU in IC and TBI in contacts after adjusting for potential confounders. The dependent variable in the regression model was TBI, which was coded as 1 for positive and 0 for negative based on the Cy-Tb test results. A *p*-value of <0.05 was considered statistically significant in the multivariable model. Data were analyzed using STATA version 17.

## 3. Results

Out of 713 social contacts participated: 358 (50.3%) were contacts of AU TB cases and 355 (49.7%) NAU cases. The mean (SD) age of contacts was 42 (16) years, i.e., 40 (16) years for the AU group and 44 (17) years for the NAU group.

IC with AU were mostly aged 45–60 years, engaged in unskilled occupations and from lower socioeconomic strata compared to non-alcohol-users. Smoking was predominantly higher among AU group, while diabetes and hypertension were more common in the NAU group. Underweight was more frequent among IC with AU (Table 1).

Among the 358 contacts of ICs with AU, 121 (33.8%; 95% CI, 29.1–38.8%) had TBI, and one (0.28%) had TB disease at baseline. Among the 355 contacts of ICs without AU, 77 (21.7%; 95% CI, 17.7–26.3%) had TBI, and none had TB disease. The proportion of TBI was significantly higher among contacts of AU ICs (61.1%) than NAU (38.9%).

Compared to contacts of NAU ICs, those in the AU group were predominantly males, from lower SES, engaged in unskilled labor and were unmarried. Smoking and AU were more common among contacts in the AU group. The AU group had more contacts with diabetes mellitus (23.2% vs. 15.2%) (Table 2).

They also had more night-time exposure to the IC and had a higher proportion of friends as social contacts Contacts of AU patients spent more time with the index case, over eight weeks together (40.8% vs. 27.8%) and all day (9.2% vs. 6.2%). Also, a higher proportion of close contacts were in the AU group (37.4%) than in the NAU group (30.1%) (Table 3). While overall AU was higher among the contacts of the patients with AU, harmful use was more common among the contacts of NAU group (31% vs. 23.9%) (Table 4).

In univariate analysis, contact type, age group, education level, socioeconomic status, body mass index (BMI), diabetes, hypertension, knowing pulmonary TB patients other than index case, family history of TB, tobacco use, smoking, alcohol use, type of contact based on the frequency of meeting and sharing food with index case had a significant association with TBI (Table 5) were considered for multivariable analysis based on their epidemiological relevance.

**Figure 2:**
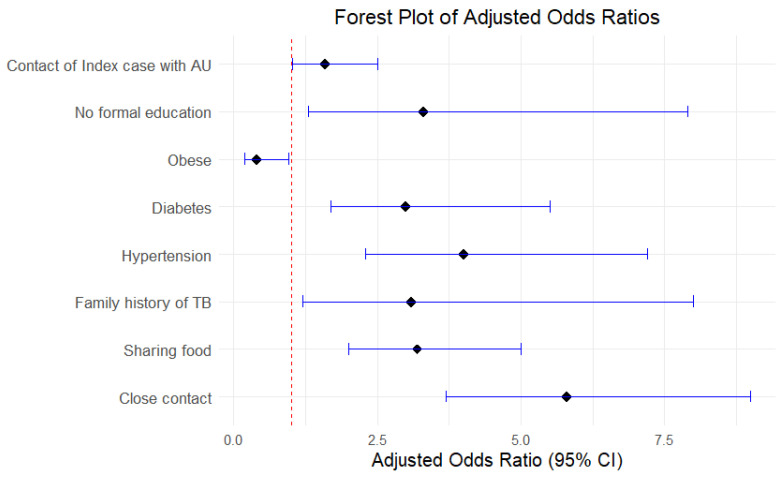
Forest Plot of Adjusted Odds Ratios for Factors Associated with TB Infection.

In the multivariable regression analysis (Table 5), numerous characteristics were identified as strongly associated with a higher likelihood of TBI among contacts (Figure 2). Contact with an IC reporting AU was significantly associated with an increased risk of TBI (aOR: 1.6, 95% CI 1.02–2.5, *p* = 0.04). Close contacts (aOR: 5.8,95% CI: 3.7–9, *p* < 0.001) were significantly more likely to acquire TBI than casual contacts and sharing food with the IC was found to be a significant predictor for TBI (aOR: 3.2, 95% CI: 2–5, *p* < 0.001). Furthermore, diabetes was identified as a significant risk factor, with patients diagnosed with diabetes having a threefold risk for TBI (aOR: 3, 95% CI: 1.7–5.5, *p* < 0.001). Similarly, hypertension was strongly associated with an increased risk of TBI (aOR:4, 95% CI: 2.3–7.2, *p* < 0.001). Participants with a familial history of tuberculosis significantly increased the probability of TBI by nearly threefold (aOR: 3.1, 95% CI: 1.2–8, *p* = 0.02). Contacts with no formal education had a much higher chance of TBI (aOR: 3.3, 95% CI: 1.3–7.9, *p* = 0.01) than those with at least a graduate degree. Additionally, obese people had a much lower chance of getting TBI (aOR: 0.4, 95% CI: 0.2–0.96, *p* = 0.036), suggesting that a higher BMI may be a protective factor. The mean variation inflation factor (VIF) was found to be 2.3, indicating no significant multicollinearity among the included independent variables.

## 4. Discussion

In this study, “social contacts” referred to individuals who shared enclosed spaces with the index case (such as friends, co-workers, relatives or alcohol-sharing partners) outside the household. In NTEP, such exposures are categorized as “close contacts,” but we used the term “social contacts” to reflect a broader range of community-based interactions. Unlike household contacts, who reside with the index case, social contacts capture diverse extra-household exposures and social mixing patterns that may influence TB transmission.

We assessed TBI among 713 contacts; 358 from AU and 358 from NAU index cases. TBI was higher among contacts in the AU group (33.8% vs. 21.7%) compared to NAU, a finding likely generalizable in Indian context. The National TB Prevalence Survey India 2019–2021 reported a 21% TBI in the general population [26], while a prior Puducherry study reported 29.6% TBI among household contacts when using a ≥10 mm Mantoux test cut-off (unpublished data) [27]. These findings indicate that extra-household transmission contributes substantially to the overall TB burden, much like household transmission.

TBI was more common among friends of PTBs, with 25% of alcohol-sharing contacts infected and 52% being friends, aligning with K Nagarajan’s findings that extra-household contacts have higher TB risk, thus highlighting the potential for transmission within social networks [24].

In present study, contacts of AU index-case were mostly with lower education, unskilled workers, and below the poverty line; they had higher TBI positivity, reflecting structural vulnerability due to poor living conditions, limited health-seeking behavior, and increased infection risk, consistent with prior research linking socioeconomic disadvantage to TB [28]. AU weakens immunity and promotes gatherings in high-risk settings like liquor shops, and combined with poor socioeconomic factors, increases TB infection susceptibility [29].

An earlier study found an inverse-relationship between AU and TBI among household contacts, likely due to less time spent at home by PTB with AU [9,27]. In South Africa, household contacts infected with the same strain as that of the PTB, may have acquired TB outside the household, since the same strain was also the most prevalent strain in the community [30], suggesting that over 75% of transmission may occur outside the household due to extensive social mixing. While household screening is economical, it has limited impact, highlighting the need for community-level screening [10,30].

This study shows that contacts of AU index cases are more vulnerable to TBI (aOR = 1.6) due to closer interactions, including prolonged time together, drinking, and food sharing. A higher proportion of contacts in the AU group were close contacts (37.4% vs. 30.1%), had contact >8 weeks (40.8% vs. 27.9%), spent nights (30.8% vs. 21.4%), and shared food (41.2% vs. 43.4%), indicating greater exposure within AU networks. TBI was 61.1% in contacts of AU group vs. 38.9% in the other, despite more harmful AU among the social contacts of NAU group. This substantial variation in infection rates strongly suggests the index case’s alcohol drinking status coupled with food sharing could have significant impacts on the transmission dynamics of TBI rather than the drinking status of the contacts. Alcohol is an immunosuppressant [31], increasing bacterial load and prolong infectiousness in people with AU, raising the risk of TB transmission. Thus, leading to poor treatment adherence and more frequent close interactions in settings like bars [13,32,33]. Food sharing, often in poorly-ventilated settings [27], may increase the transmission risk and often coincide with alcohol consumption. The findings suggest AU as a key modifiable risk factor in TB transmission.

Close contact was the strongest predictor of TBI (aOR = 5.8), reinforcing that duration and proximity drive transmission, particularly in AU networks. A recent study also proposed scoring contact duration and frequency to improve extra-household TB screening efforts [16].

These findings show that diabetes and hypertension were associated with a higher risk of TBI. Other studies show that, these comorbidities were more common in the AU group, suggesting alcohol may worsen or contribute to these conditions, further increasing TBI risk. Additionally, studies have found that diabetes and hypertension are more prevalent among individuals with TB infection, indicating that these comorbidities may further compound the risk [34,35,36]. Targeted screening and TB preventive treatment in groups with chronic diseases such as diabetes, hypertension and heavy alcohol use may aid TB control.

TBI was also more likely among individuals with no formal education. Low literacy is often linked to unskilled, low-paying jobs in informal sectors where AU is common, increasing transmission risk. Public health interventions in low-literacy communities could reduce TB spread [37].

Obesity was linked to lower TBI odds (aOR: 0.4; 95% CI: 0.2–0.96), in line with studies showing an inverse relationship between BMI and TB risk. Various studies have shown that higher BMI reduces both the likelihood of TB and progression to active disease, with each unit increase in BMI linked to a 2% decline in TB incidence [38,39,40].

## 5. Conclusions and Recommendations

The study found a significantly higher burden of TB infection among social contacts of alcohol-using index cases compared to non-alcohol-using cases, empasizing alcohol use as an important factor in TB transmission dynamics. Key predictors of TBI included close contact with the index case, food sharing, diabetes, hypertension, lack of formal education, and familial history of TB, while higher BMI appeared protective.

This study underscores the role of AU in TB transmission and the need to expand contact tracing beyond households. Risk-based screening under the National TB Program could be more effective, particularly for socially vulnerable groups, and in high-risk settings, including bars, workplaces, and other social gathering spots. Thus, helps in the early identification of undiagnosed cases and enhance overall coverage of TB control efforts. Psychological counseling at treatment initiation can reduce stigma and encourage social contact disclosure, while peer involvement through TB survivors with alcohol use history can facilitate tracing and engaging high-risk individuals. These identified contacts can then be screened for TB infection and further tested for TB disease. Depending on the results, they can either be given TB preventive therapy or be directed to the treatment for active TB. To improve adherence, shorter TPT regimens like 3HP could be considered for these groups, particularly for individuals who use alcohol and are less likely to seek treatment. At the same time, places where patients often gather like tea shops, workplaces, or bars can be identified, and targeted active screening can be carried out in those areas of congregations using mobile screening units. TB health workers and caregivers can help reduce stigma, improve health seeking behaviour in them, and decrease the chances of patients dropping out. Liquor vendors can be trained to recognize TB symptoms and refer symptomatic individuals to health services. To raise public awareness and promote self-referral, educational posters highlighting key TB symptoms and health helpline numbers can be displayed in high-risk spots such as bars and liquor stores. Future research could test the feasibility and acceptability of training liquor vendors to recognize TB symptoms, ensuring the approach is practical and non-stigmatizing.

## 6. Limitations

Relying on patient-reported contacts may have introduced recall bias and underreporting. Social desirability bias may have led to misreporting of certain information, such as alcohol consumption or comorbidities like diabetes. This could have introduced misclassification bias in this study. Moreover, the cross-sectional design of this study limits the ability to establish causal relationships between alcohol use and TB infection.

## Figures and Tables

**Table 1:** Sociodemographic characteristics of Index case.

Characteristics	IC with AU (N1 = 45)		IC without AU (N2 = 61)		Total IC (N = 106)	
	n	%	n	%	n	%
**Age groups (years)** 19–30 31–45 46–60 >60	3 12 27 3	6.7 26.7 60 6.7	5 10 21 25	8.2 16.4 34.4 41	8 22 48 28	7.6 20.8 45.2 26.4
**Area of residence** Rural Urban	5 40	11.1 88.9	9 52	14.8 85.2	14 92	13.2 86.8
**Religion** Hindu Christian Muslim	41 3 1	91.1 6.7 2.2	55 2 4	90.2 3.3 6.6	96 5 5	90.6 4.7 4.7
**Education** Illiterate Primary Secondary Higher secondary Graduate	4 14 17 3 7	8.9 31.1 37.8 6.7 15.6	4 13 30 3 11	6.6 21.3 49.2 4.9 18	8 27 47 6 18	7.5 25.5 44.4 5.7 16.9
**Occupation** Unskilled Unemployed Skilled Student Professional	29 0 11 0 5	64.4 0 24.4 0 11.1	25 3 23 1 9	41 4.9 37.7 1.6 14.8	54 3 34 1 14	50.9 2.8 32.2 0.9 13.2
**SES** APL BPL	5 40	11.1 88.9	18 43	29.5 70.5	23 83	21.7 78.3
**Marital status** Married Unmarried Separated/Widow	39 5 1	86.7 11.1 2.2	52 90	85.2 14.80	91 14 1	85.8 13.2 0.94
**Type of TB** New Recurrent	40 5	88.9 11.1	55 6	90.2 9.8	95 11	89.6 10.4
**BCG Scar** Yes	43	95.6	54	88.5	97	91.5
**Smoking** Yes	7	15.6	2	3.3	9	8.5
**Chronic diseases** Yes	18	40	40	65.6	58	54.7
**Diabetes** Yes	15	33.3	34	55.7	49	46.2
**Hypertension** Yes	4	8.9	16	26.2	20	18.8
**Body mass index (kg/m^2^)** Underweight (<18.5) Normal (18.5–22.9) Overweight (23–24.9) Obese (>25)	25 15 4 1	55.6 33.3 8.9 2.2	25 29 6 1	41 47.5 9.8 1.6	50 44 10 2	47.2 41.5 9.4 1.9
**Contact History of TB** Yes	17	37.8	16	26.2	33	31.1
**Smear grade** Scanty 1+ 2+ 3+	4 21 13 7	8.9 46.7 28.9 15.6	14 29 10 8	23 47.5 16.4 13.1	18 50 23 15	16.9 47.2 21.7 14.2

IC = Index case, AU = Alcohol Use, APL = Above Poverty Line, BPL = Below Poverty Line, Skilled worker = Driver, cook, electrician, barber, carpenter, Unskilled worker = labour, construction worker, painter, shopkeeper, street vendor, scrap picker, security, Chronic diseases = CVD, Hypothyroidism, Hyperthyroidism, CKD, Epilepsy, Stroke, Diabetes, Hypertension.

**Table 2:** Sociodemographic characteristics of Social Contacts of Index Case.

Characteristics	Social Contacts of IC with AU (N = 358)		Social Contacts of IC without AU (N = 355)		Total Social Contacts (N = 713)	
	n	%	n	%	n	%
**Age** ≤18 19–30 31–45 46–60 >60	27 79 103 117 32	7.5 22.1 28.8 32.7 8.9	20 58 121 92 64	5.6 16.3 34.2 25.9 18	47 137 224 209 96	6.6 19.2 31.4 29.3 13.5
**Area of residence** Rural Urban	60 298	16.8 83.2	61 294	17.2 82.8	121 592	16.9 83.1
**Gender** Female Male	156 202	43.6 56.4	170 185	47.9 52.1	326 387	45.7 54.3
**Religion** Hindu Christian Muslim	316 38 4	88.3 10.6 1.1	316 21 18	89 5.9 5.1	632 59 22	88.6 8.3 3.1
**Education** No formal education Primary Secondary Higher secondary Graduate	48 82 134 35 59	13.4 22.9 37.4 9.8 16.5	55 67 115 48 70	15.5 18.9 32.4 13.5 19.7	103 149 249 83 129	14.4 20.9 34.9 11.6 18.2
**Occupation** Unskilled Unemployed Skilled Student Professional Retired	153 81 75 28 20 1	42.7 22.6 20.9 7.8 5.7 0.3	125 93 76 29 23 9	35.2 26.2 21.4 8.2 6.5 2.5	278 174 151 57 43 10	38.9 24.4 21.2 7.9 6 1.6
**Socioeconomic Status** APL BPL	65 293	18.2 81.8	148 207	41.7 58.3	213 500	29.8 70.2
**Marital status** Married Unmarried Separated/Widow	278 79 1	77.6 22.1 0.3	288 60 7	81.1 16.9 2	566 139 8	79.4 19.5 1.1
**BCG Scar** Yes	320	89.4	318	89.6	638	89.5
**Smoking Status** Yes	75	20.9	31	8.7	106	14.9
**Alcohol use** Yes	138	38.5	58	16.3	196	27.5
**Spent night with index case** Yes	110	30.8	76	21.4	186	26.1
**Share food** Yes	147	41.2	154	43.4	301	42.2
**Presence of Chronic Diseases** Yes	122	34.1	103	29	225	31.6
**Diabetes** Yes	83	23.2	54	15.2	137	19.2
**Hypertension** Yes	82	22.9	77	21.7	159	22.3
**Body Mass Index** Underweight (<18.5) Normal (18.5–22.9) Overweight (23–24.9) Obese (>25)	50 131 44 133	14 36.6 12.2 37.2	34 128 45 148	9.6 36.1 12.6 41.7	84 259 89 281	11.8 36.3 12.4 39.5

IC = Index case, AU = Alcohol Use, APL = Above Poverty Line, BPL = Below Poverty Line, Skilled worker = Driver, cook, electrician, barber, carpenter, Unskilled worker = labour, construction worker, painter, shopkeeper, street vendor, scrap picker, Chronic diseases = Hypertension, Diabetes Mellitus, CVD, Hypothyroidism, hyperthyroidism, CKD, Epilepsy, Arthritis, Asthma, Pancreatitis.

**Table 3:** Epidemiological and social relationship between index case and social contacts.

Characteristics	Social Contacts of IC with AU (N = 358)		Social Contacts of IC without AU (N = 355)		Total Social Contacts (N = 713)	
	n	%	n	%	n	%
**Relation** Extended family Friend Neighbour Relative Workplace contact	37 63 48 109 101	10.3 17.6 13.4 30.4 28.3	71 47 23 109 105	20 13.2 6.5 30.7 29.6	108 110 71 218 206	15.1 15.4 9.9 30.6 29
**Past TB history** Yes	2	0.6	3	0.8	5	0.7
**Knows TB patient other than index case** Yes	38	10.6	39	11	77	10.8
**Family history of TB** Yes	25	7	24	6.8	49	6.8
**Family history of death due to TB** Yes	3	0.8	7	2	10	1.4
**Type of contact** Casual contact Close contact	224 134	62.6 37.4	248 107	69.9 30.1	472 241	66.2 33.8
**Duration of knowing index case** <12 years ≥12 years	199 159	55.6 44.4	155 200	43.7 56.3	354 359	49.6 50.3
**Weeks spend with index case** <4 weeks 4–8 weeks >8 weeks	75 137 146	20.9 38.3 40.8	92 164 99	25.9 46.2 27.9	167 301 245	23.4 42.2 34.4
**Times in a week** 3+ times/week Everyday/week	219 139	61.2 38.8	224 131	63.1 36.9	443 270	62.1 37.9
**Hours in a week** 2–4 h/week 4+ h/week All day	172 153 33	48 42.8 9.2	201 132 22	56.6 37.2 6.2	373 285 55	52.3 39.9 7.8
**Spend night with index case** Yes	110	30.8	76	21.4	186	26.1
**Share food** Yes	147	41.2	154	43.4	301	42.2

**Table 4:** Alcohol use among social contacts of index case.

Variables	Social Contacts of IC with AU (N = 138)		Social Contacts of IC without AU (N = 58)	
	n	%	n	%
AUDIT score Low risk (0–3) Risky (4–9) Harmful (10–13) Severe (14+)	3 47 33 55	2.1 34.1 23.9 39.9	1 16 18 23	1.7 27.6 31 39.7
Drink in arrack shop Yes No	106 32	76.8 23.2	41 17	70.6 29.4
Share alcohol with IC Yes	75	54.3		-
Frequency of drink with IC 1–2 times/week 3+ times/week Everyday Less than once/week	26 23 22 4	34.6 30.6 29.4 5.4		-
Share glass Yes No	3 72	4 96		-

**Table 5:** Factors associated with Tuberculosis Infection among Social Contacts of Tuberculosis Index cases.

Variables	Total	TBI Positive		TBI Negative		Unadjusted Odds Ratio (95%CI)	Adjusted Odds Ratio (95%CI)	Adjusted *p* Value
	N	n	%	n	%			
**Contact Type** Index case with AU Index case without AU	357 355	121 77	61.6 38.9	236 278	45.9 54.1	1.9 (1.3–2.5) (ref)	1.6 * (1.02–2.5) (ref)	0.04
**Age** ≤18 19–30 31–45 46–60 >60	47 137 224 208 96	8 33 61 64 32	4 16.6 30.8 32.4 16.2	39 104 163 144 64	7.6 20.2 31.7 28 12.5	(ref) 1.6 (0.6–3.6) 1.8 (0.8–4.1) 2.2 (0.9–4.8) 2.4 (1.02–5.8)	(ref) 1.3 (0.5–4) 1 (0.4–3) 0.5 (0.2–1.6) 0.5 (0.1–1.8)	0.5 0.8 0.3 0.3
**Education** No formal education School level Graduate level	103 480 129	41 139 18	20.7 70.2 9.1	62 341 111	12 66.4 21.6	4 (2.2–7.7) 2.5 (1.4–4.3) (ref)	3.3 (1.3–7.7) 1.8 (0.9–3.5) (ref)	0.01 0.08
**Occupation** Unskilled Unemployed Skilled Student Professional Retired	278 173 151 57 43 10	93 48 42 5 9 1	47 24.2 21.2 2.5 4.6 0.5	185 125 109 52 34 9	36 24.3 21.2 10.1 6.6 1.8	1.9 (0.8–4.1) 1.5 (0.6–3.2) 1.5 (0.6–3.3) 0.4 (0.1–1.2) (ref) 0.4 (0.05–3.8)	Not included in model	
**Area of residence** Rural Urban	120 592	39 159	19.7 80.3	81 433	15.8 84.2	1.3 (0.8–2) (ref)	Not included in model	
**Gender** Female Male	325 387	84 114	42.4 57.6	241 273	46.9 53.1	0.8 (0.6–1.2) (ref)	Not included in model	
**Religion** Hindu Christian Muslim	631 59 22	172 19 7	86.8 9.6 3.6	459 40 15	89.3 7.8 2.9	(ref) 1.3 (0.7–2.2) 1.2 (0.5–3)	Not included in model	
**Socioeconomic Status** APL BPL	213 499	37 161	18.7 81.3	176 338	34.2 65.8	(ref) 2.3 (1.5–3.4)	*Not included in model*	
**BMI** Underweight Normal Overweight Obese	84 258 89 281	39 68 30 61	19.7 34.3 15.2 30.8	45 190 59 220	8.8 36.9 11.5 42.8	(ref) 0.4 (0.3–0.7) 0.6 (0.3–1.1) 0.3 (0.2–0.5)	(ref) 0.5 (0.3–1.1) 1 (0.4–2.4) 0.5 * (0.2–0.95)	0.1 0.8 0.036
**Diabetes** Yes No	137 575	72 126	36.4 63.6	65 449	12.6 87.4	3.9 (2.7–5.8) (ref)	3 * (1.7–5.5) (ref)	<0.001
**Hypertension** Yes No	159 553	81 117	40.9 59.1	77 437	15 85	3.9 (2.7–5.7) (ref)	4 * (2.3–7) (ref)	<0.001
**Knowing person with TB other than index case** Yes No	77 635	41 157	20.7 79.3	36 478	7 93	3.5 (2.1–5.6) (ref)	2.3 (0.9–5.1) (ref)	0.05
**Family history of TB** Yes No	49 663	27 171	13.6 86.4	22 492	4.3 95.7	3.5 (1.9–6.4) (ref)	3.1 * (1.2–8) (ref)	0.02
**Smoking** Yes No	106 606	59 139	29.8 70.2	47 467	9.1 90.9	4.2 (2.7–6.5) (ref)	1.8 (0.9–3.7) (ref)	0.08
**Alcohol use** Yes No	196 516	91 107	46 54	105 409	20.4 79.6	3.3 (2.3–4.7) (ref)	1.2 (0.7–2.2) (ref)	0.5
**Sharing food** Yes No	301 412	131 67	66.2 33.8	170 344	33.1 66.9	3.9 (2.8–5.6)	3.2 * (2–5) (ref)	<0.001
**Type of contact based on frequency of meeting** Casual contact Close contact	472 241	70 128	35.4 64.6	401 113	78 22	(ref) 6.5 (4.5–9.3)	(ref) 5.8 * (3.7–9)	<0.001

* The variables such as occupation, area of residence, religion, gender, and socioeconomic status were excluded from the multivariable model due to either a lack of statistical significance in univariate analysis (*p* > 0.20) or concerns related to multicollinearity (mean VIF = 2.3). Univariate results correspond to chi-square tests.

## Data Availability

The data supporting the findings of this study are available from the corresponding author upon reasonable request.

## Abbreviations

The following abbreviations are used in this manuscript:

aOR	Adjusted Odds Ratio
AU	Alcohol use
AUD	Alcohol use disorder
BMI	Body Mass Index
IC	Index case
NAU	Non-alcohol use
PTB	Persons with Tuberculosis
TB	Tuberculosis
TBI	Tuberculosis Infection
